# *catena*-Poly[[tricarbonyl-1κ^3^ *C*-(1η^5^-cyclo­penta­dien­yl)lead(II)molyb­denum(0)](*Mo*—*Pb*)-μ_3_-acetato-2′:2:2′′κ^4^ *O*:*O*,*O*′:*O*′]

**DOI:** 10.1107/S1600536812024737

**Published:** 2012-06-02

**Authors:** Eberhard Gerdes, Kurt Merzweiler

**Affiliations:** a Institut für Chemie, Naturwissenschaftliche Fakultät II, Martin-Luther-Universität Halle-Wittenberg, Kurt-Mothes-Strasse 2, 06120 Halle, Germany

## Abstract

In the title compound, [MoPb(C_5_H_5_)(CH_3_COO)(CO)_3_], the Pb^II^ atom is coordinated pyramidally *via* the Mo^0^ atom of a {Cp(CO)_3_Mo} (Cp = cyclo­penta­dien­yl) fragment [Pb—Mo = 3.0589 (5) Å] and a chelating acetate (Ac) group. Additionally, the [{Cp(CO)_3_Mo}PbAc] units are linked along [100] by Pb—O(acetate) inter­actions, giving a ladder-type polymeric structure composed of PbCO_2_ and Pb_2_O_2_ four-membered rings. The {Cp(CO)_3_Mo} units attached to the Pb^II^ atom occupy terminal positions at opposite sides of the slightly puckered lead acetate chain. The angle between the Pb—Mo bond vector and the central chain plane is 67.8 (2)°.

## Related literature

For organometallic compounds containing Pb—Mo bonds, see: Kubicki *et al.* (1984 ▶); Hitchcock *et al.* (1987 ▶); Pu *et al.* (2000 ▶); Campbell *et al.* (2002 ▶); Yong *et al.* (2005**a* ▶,b* ▶); Alonso *et al.* (2010 ▶). For lead(II) carboxyl­ates with ladder-type structures, see: Rajaram & Rao (1982 ▶); Dai *et al.* (2009 ▶).
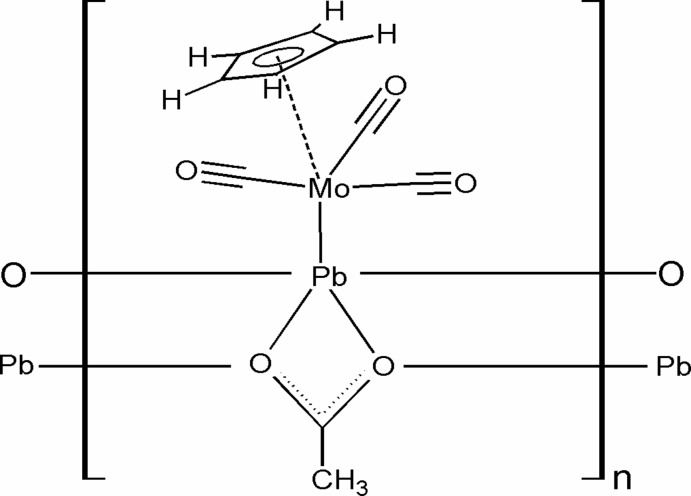


## Experimental

### 

#### Crystal data

[MoPb(C_5_H_5_)(C_2_H_3_O_2_)(CO)_3_]
*M* *_r_* = 511.29
Monoclinic, 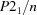

*a* = 7.4813 (6) Å
*b* = 14.9431 (12) Å
*c* = 11.3323 (9) Å
β = 98.660 (9)°
*V* = 1252.44 (17) Å^3^
*Z* = 4
Mo *K*α radiation
μ = 14.43 mm^−1^
*T* = 220 K
0.19 × 0.19 × 0.08 mm

#### Data collection

Stoe IPDS I diffractometer
Absorption correction: numerical (*IPDS Program Package*; Stoe & Cie, 1999 ▶) *T*_min_ = 0.112, *T*_max_ = 0.263
9600 measured reflections
2417 independent reflections
2130 reflections with *I* > 2σ(*I*)
*R*_int_ = 0.066

#### Refinement

*R*[*F* ^2^ > 2σ(*F* ^2^)] = 0.024
*wR*(*F* ^2^) = 0.056
*S* = 1.03
2417 reflections
154 parameters
H-atom parameters constrained
Δρ_max_ = 1.29 e Å^−3^
Δρ_min_ = −1.15 e Å^−3^

### 

Data collection: *IPDS Program Package* (Stoe & Cie, 1999 ▶); cell refinement: *IPDS Program Package*; data reduction: *IPDS Program Package*; program(s) used to solve structure: *SHELXS97* (Sheldrick, 2008 ▶); program(s) used to refine structure: *SHELXL97* (Sheldrick, 2008 ▶); molecular graphics: *DIAMOND* (Brandenburg, 2009 ▶); software used to prepare material for publication: *publCIF* (Westrip, 2010 ▶).

## Supplementary Material

Crystal structure: contains datablock(s) I, global. DOI: 10.1107/S1600536812024737/wm2639sup1.cif

Structure factors: contains datablock(s) I. DOI: 10.1107/S1600536812024737/wm2639Isup2.hkl

Additional supplementary materials:  crystallographic information; 3D view; checkCIF report

## Figures and Tables

**Fig. 1.**
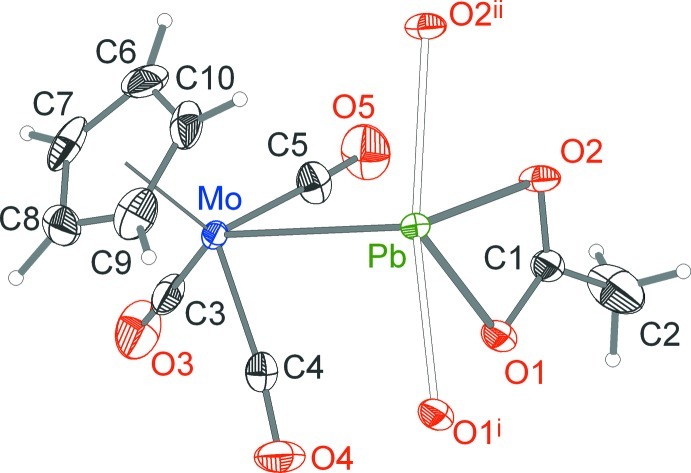
Coordination around the lead atom in the structure of compound (I). The asymmetric unit is shown with filled bonds. Thermal ellipsoids are drawn at the 50% probability level. [Symmetry codes: (i): 1 - x, 1 - y, 1 - z, (ii): -x, -y + 1, -z + 1.]

**Fig. 2.**
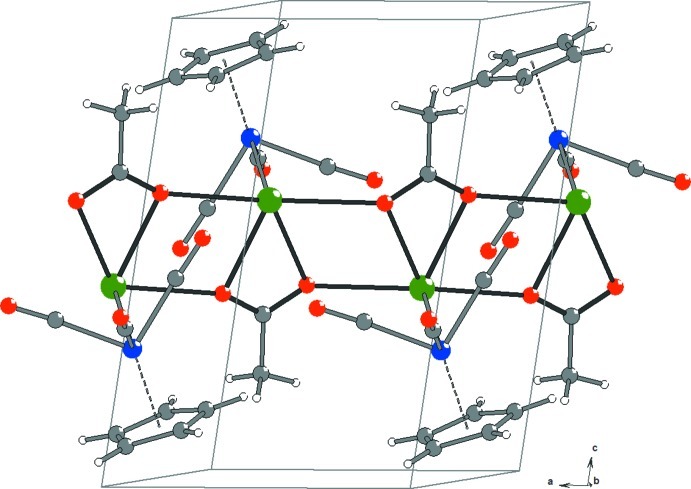
Part of the ladder type chain structure in compound (I).

## supplementary crystallographic information

### Comment

In the title compound, [Mo(C_5_H_5_)(CO)_3_Pb(CH_3_COO)], (I), the lead(II) atom is coordinated by a {Cp(CO)_3_Mo} fragment and a chelating acetate group to give a pyramidal PbMoO_2_ unit with lead at its apex (Fig. 1). The Pb—Mo distance of 3.0589 (5) Å is slightly longer than the values that have been observed in other organometallic lead(II) complexes, like [{Cp*(CO)_3_Mo}_2_Pb(thf)] (2.989 and 3.019 Å), [{Cp*(CO)_3_Mo}_2_Pb_2_] (2.935 and 2.989 Å; Hitchcock *et al.*, 1987) and [{Cp(CO)_3_MoPb*R*] (2.986 Å, *R* = 2,6-bis(2,4,6-triisopropylphenyl)phenyl; Pu *et al.*, 2000). Complexes containing polynuclear lead clusters as ligands display Pb—Mo distances within a similar range, *e.g.* [Pb_9_{Mo(CO)_3_}]^4-^ (2.962–3.241 Å; Yong *et al.*, 2005*a*; 2.985–3.084 Å; Campbell *et al.*, 2002), [Pb_5_{Mo(CO)_3_}_2_]^2-^ (3.001–3.093 Å; Yong *et al.*, 2005*b*). In the case of the Pb(IV) derivatives [{Cp_2_MoH}_2_PbAc_2_] (Kubicki *et al.*, 1984) and [MCp{P(O)*R**}(CO)2(PbPh_3_)] (*R** = 2,4,6-C_6_H_2_*^t^*Bu_3_) (Alonso *et al.*, 2010) considerably shorter Pb—Mo distances of 2.808 Å and 2.8845 Å, respectively, have been observed.

The acetate group forms two Pb—O bonds with 2.491 (4) and 2.522 (4) Å to give a PbCO_2_ chelate ring. Additionally, the acetate group interacts with two neighbouring lead(II) atoms with Pb—O distances of 2.703 (4) and 2.752 (4) Å. Due to this µ_3_-κ^4^-*O*,*O*:*O*':*O*' coordination mode a chain structure along [100] with alternating PbCO_2_ and Pb_2_O_2_ rings is formed. The Pb_2_O_2_ four-membered rings display exact planarity (crystallographic 1 symmetry) and the PbCO_2_ rings are nearly planar (deviation from the rms plane = 0.009 (4) Å). The interplanar angle between the PbCO_2_ rings and the Pb_2_O_2_ units is 29.7 (2)° and consequently a slightly puckered ladder chain is formed (Fig. 2). Similar ladder chain motifs have been observed in other Pb(II) carboxylate compounds like lead(II) diacetate trihydrate (Rajaram & Rao, 1982) and lead(II) bis (3-methylbenzoate) trihydrate (Dai *et al.*, 2009).

### Experimental

The title compound (I) was synthesized by two different routes:

Method A

2.68 g of Na{Cp(CO)_3_Mo} (10 mmol) were dissolved in 50 ml of thf and cooled to 195 K. To this solution 1.63 g (5 mmol) of lead(II) acetate in 50 ml thf were added. The reaction mixture was stirred at 195 K for one hour and then slowly warmed up to 248 K. The originally pale yellow solution changed to blue green. After one day the reaction mixture was filtered over celite and the filtrated was layered with *n*-heptane. After some hours orange crystals of [{Cp(CO)_3_Mo}Pb(Ac)] formed at the phase boundary.

Yield: 0.92 g (36%)

Method B

1.4 g of Pb(II) acetate trihydrate (3.73 mmol) were dissolved in 20 ml of water and conc. acetic acid was added to adjust the pH value to approx. 4. After addition of a solution of 1.0 g (3.73 mmol) of Na{Cp(CO)_3_Mo} in 20 ml of H_2_O an orange coloured precipitate formed. The precipitate was filtered off and washed with water. After drying *in vacuo*, the residue was extracted with thf to give an orange solution which is layered with *n*-heptane. Within two days bright orange crystals of [{Cp(CO)_3_Mo}Pb(Ac)] formed.

Yield: 0.5 g (26%)

Analysis, calculated for C_10_H_8_MoO_5_Pb: C 23.5, H 1.58%, found: C 22.2, H 1.49%

### Refinement

H atoms were placed in calculated positions with C—H distances of 0.97 Å for the CH_3_ group and 0.94 Å for the Cp group, *U*_iso_(H) = 1.2 *U*_eq_(C). The highest and lowest remaining electron densities were found 1.93 Å from Pb and 0.91 Å, respectively, from the same atom.

### Crystal data

**Table:**

[MoPb(C_5_H_5_)(C_2_H_3_O_2_)(CO)_3_]	*F*(000) = 928
*M**_r_* = 511.29	*D*_x_ = 2.712 Mg m^−^^3^
Monoclinic, *P*2_1_/*n*	Mo *K*α radiation, λ = 0.71073 Å
Hall symbol: -P 2yn	Cell parameters from 8000 reflections
*a* = 7.4813 (6) Å	θ = 2.3–25.9°
*b* = 14.9431 (12) Å	µ = 14.43 mm^−^^1^
*c* = 11.3323 (9) Å	*T* = 220 K
β = 98.660 (9)°	Plate, orange
*V* = 1252.44 (17) Å^3^	0.19 × 0.19 × 0.08 mm
*Z* = 4	

### Data collection

**Table:**

Stoe IPDS I diffractometer	2417 independent reflections
Radiation source: fine-focus sealed tube	2130 reflections with *I* > 2σ(*I*)
Graphite monochromator	*R*_int_ = 0.066
φ oscillation scans	θ_max_ = 25.9°, θ_min_ = 2.3°
Absorption correction: numerical (*IPDS Program Package*; Stoe & Cie, 1999)	*h* = −9→9
*T*_min_ = 0.112, *T*_max_ = 0.263	*k* = −18→18
9600 measured reflections	*l* = −13→13

### Refinement

**Table:**

Refinement on *F*^2^	Primary atom site location: structure-invariant direct methods
Least-squares matrix: full	Secondary atom site location: difference Fourier map
*R*[*F*^2^ > 2σ(*F*^2^)] = 0.024	Hydrogen site location: inferred from neighbouring sites
*wR*(*F*^2^) = 0.056	H-atom parameters constrained
*S* = 1.03	*w* = 1/[σ^2^(*F*_o_^2^) + (0.0275*P*)^2^] where *P* = (*F*_o_^2^ + 2*F*_c_^2^)/3
2417 reflections	(Δ/σ)_max_ = 0.001
154 parameters	Δρ_max_ = 1.29 e Å^−^^3^
0 restraints	Δρ_min_ = −1.15 e Å^−^^3^

### Special details

**Table:**

Geometry. All e.s.d.'s (except the e.s.d. in the dihedral angle between two l.s. planes) are estimated using the full covariance matrix. The cell e.s.d.'s are taken into account individually in the estimation of e.s.d.'s in distances, angles and torsion angles; correlations between e.s.d.'s in cell parameters are only used when they are defined by crystal symmetry. An approximate (isotropic) treatment of cell e.s.d.'s is used for estimating e.s.d.'s involving l.s. planes.
Refinement. Refinement of *F*^2^ against ALL reflections. The weighted *R*-factor *wR* and goodness of fit *S* are based on *F*^2^, conventional *R*-factors *R* are based on *F*, with *F* set to zero for negative *F*^2^. The threshold expression of *F*^2^ > σ(*F*^2^) is used only for calculating *R*-factors(gt) *etc*. and is not relevant to the choice of reflections for refinement. *R*-factors based on *F*^2^ are statistically about twice as large as those based on *F*, and *R*- factors based on ALL data will be even larger.

### Fractional atomic coordinates and isotropic or equivalent isotropic displacement parameters (Å^2^)

**Table:**

	*x*	*y*	*z*	*U*_iso_*/*U*_eq_	
C1	0.2861 (8)	0.5561 (4)	0.6488 (5)	0.0223 (12)	
C2	0.3210 (12)	0.5900 (6)	0.7737 (6)	0.057 (2)	
H3	0.4449	0.6109	0.7915	0.068*	
H2	0.3018	0.5422	0.8282	0.068*	
H1	0.2392	0.6391	0.7826	0.068*	
C3	0.2737 (9)	0.7886 (4)	0.3061 (5)	0.0283 (13)	
C4	0.4537 (8)	0.6461 (4)	0.3283 (5)	0.0223 (12)	
C5	0.0974 (8)	0.6837 (4)	0.4177 (5)	0.0259 (13)	
C6	−0.0802 (9)	0.6429 (5)	0.1399 (6)	0.0406 (17)	
H4	−0.1923	0.6482	0.1672	0.049*	
C7	0.0154 (10)	0.7132 (4)	0.0927 (5)	0.0385 (17)	
H5	−0.0196	0.7736	0.0847	0.046*	
C8	0.1749 (10)	0.6743 (5)	0.0599 (5)	0.0378 (16)	
H6	0.2632	0.7048	0.0247	0.045*	
C9	0.1782 (10)	0.5829 (5)	0.0891 (5)	0.0384 (16)	
H7	0.2687	0.5415	0.0777	0.046*	
C10	0.0208 (10)	0.5650 (4)	0.1386 (5)	0.0357 (16)	
H8	−0.0113	0.5089	0.1666	0.043*	
O1	0.4139 (5)	0.5565 (3)	0.5865 (3)	0.0244 (9)	
O2	0.1338 (5)	0.5251 (3)	0.6061 (4)	0.0246 (9)	
O3	0.3190 (8)	0.8626 (3)	0.3264 (4)	0.0491 (13)	
O4	0.6102 (6)	0.6423 (3)	0.3575 (4)	0.0376 (11)	
O5	0.0370 (7)	0.7031 (3)	0.5033 (4)	0.0465 (13)	
Mo	0.19661 (6)	0.66595 (3)	0.26799 (4)	0.01695 (12)	
Pb	0.22870 (3)	0.489761 (12)	0.406981 (17)	0.01569 (8)	

### Atomic displacement parameters (Å^2^)

**Table:**

	*U*^11^	*U*^22^	*U*^33^	*U*^12^	*U*^13^	*U*^23^
C1	0.020 (3)	0.028 (3)	0.019 (3)	−0.002 (2)	0.003 (2)	0.000 (2)
C2	0.055 (5)	0.084 (6)	0.034 (4)	−0.037 (5)	0.014 (4)	−0.021 (4)
C3	0.031 (4)	0.024 (3)	0.026 (3)	0.002 (3)	−0.007 (3)	0.006 (2)
C4	0.029 (4)	0.018 (3)	0.020 (3)	0.004 (2)	0.006 (3)	0.000 (2)
C5	0.033 (4)	0.018 (3)	0.029 (3)	0.006 (2)	0.011 (3)	0.002 (2)
C6	0.021 (4)	0.069 (5)	0.028 (3)	−0.007 (3)	−0.007 (3)	−0.003 (3)
C7	0.053 (5)	0.030 (3)	0.024 (3)	0.006 (3)	−0.020 (3)	−0.002 (3)
C8	0.035 (4)	0.063 (5)	0.015 (3)	−0.016 (3)	0.003 (3)	0.000 (3)
C9	0.051 (5)	0.040 (4)	0.022 (3)	0.003 (3)	−0.003 (3)	−0.010 (3)
C10	0.050 (5)	0.034 (4)	0.019 (3)	−0.018 (3)	−0.011 (3)	0.004 (2)
O1	0.019 (2)	0.032 (2)	0.023 (2)	−0.0025 (17)	0.0073 (18)	0.0014 (16)
O2	0.013 (2)	0.031 (2)	0.029 (2)	−0.0035 (17)	0.0006 (17)	−0.0033 (17)
O3	0.069 (4)	0.023 (2)	0.048 (3)	−0.013 (2)	−0.013 (3)	0.004 (2)
O4	0.018 (3)	0.040 (3)	0.054 (3)	−0.0005 (19)	0.003 (2)	0.001 (2)
O5	0.053 (3)	0.049 (3)	0.042 (3)	0.017 (2)	0.022 (3)	−0.001 (2)
Mo	0.0173 (2)	0.0174 (2)	0.0154 (2)	−0.00063 (18)	−0.00030 (18)	0.00218 (16)
Pb	0.01277 (13)	0.01757 (12)	0.01657 (11)	0.00013 (8)	0.00171 (8)	0.00066 (7)

### Geometric parameters (Å, º)

**Table:**

C1—O2	1.258 (7)	C7—Mo	2.339 (6)
C1—O1	1.272 (7)	C7—H5	0.9400
C1—C2	1.488 (9)	C8—C9	1.405 (10)
C2—H3	0.9700	C8—Mo	2.342 (6)
C2—H2	0.9700	C8—H6	0.9400
C2—H1	0.9700	C9—C10	1.404 (10)
C3—O3	1.168 (7)	C9—Mo	2.363 (6)
C3—Mo	1.951 (6)	C9—H7	0.9400
C4—O4	1.169 (7)	C10—Mo	2.361 (6)
C4—Mo	1.965 (6)	C10—H8	0.9400
C5—O5	1.166 (7)	O1—Pb	2.491 (4)
C5—Mo	1.970 (5)	O1—Pb^i^	2.752 (4)
C6—C10	1.390 (10)	O2—Pb	2.522 (4)
C6—C7	1.420 (10)	O2—Pb^ii^	2.703 (4)
C6—Mo	2.368 (7)	Mo—Pb	3.0589 (5)
C6—H4	0.9400	Pb—O2^ii^	2.703 (4)
C7—C8	1.426 (10)	Pb—O1^i^	2.752 (4)
			
O2—C1—O1	120.1 (5)	Pb—O2—Pb^ii^	110.43 (15)
O2—C1—C2	120.7 (5)	C3—Mo—C4	79.8 (2)
O1—C1—C2	119.2 (6)	C3—Mo—C5	79.6 (2)
C1—C2—H3	109.5	C4—Mo—C5	101.3 (2)
C1—C2—H2	109.5	C3—Mo—C7	91.1 (2)
H3—C2—H2	109.5	C4—Mo—C7	138.9 (2)
C1—C2—H1	109.5	C5—Mo—C7	116.5 (3)
H3—C2—H1	109.5	C3—Mo—C8	98.4 (2)
H2—C2—H1	109.5	C4—Mo—C8	105.9 (2)
O3—C3—Mo	178.6 (5)	C5—Mo—C8	151.9 (3)
O4—C4—Mo	172.8 (5)	C7—Mo—C8	35.5 (2)
O5—C5—Mo	173.3 (5)	C3—Mo—C10	148.9 (2)
C10—C6—C7	108.1 (6)	C4—Mo—C10	123.0 (2)
C10—C6—Mo	72.6 (4)	C5—Mo—C10	112.0 (3)
C7—C6—Mo	71.3 (4)	C7—Mo—C10	57.9 (2)
C10—C6—H4	125.9	C8—Mo—C10	57.4 (2)
C7—C6—H4	125.9	C3—Mo—C9	131.3 (2)
Mo—C6—H4	121.8	C4—Mo—C9	98.4 (2)
C6—C7—C8	106.6 (6)	C5—Mo—C9	146.0 (3)
C6—C7—Mo	73.6 (4)	C7—Mo—C9	58.6 (2)
C8—C7—Mo	72.4 (4)	C8—Mo—C9	34.8 (2)
C6—C7—H5	126.7	C10—Mo—C9	34.6 (2)
C8—C7—H5	126.7	C3—Mo—C6	118.3 (3)
Mo—C7—H5	119.3	C4—Mo—C6	155.7 (2)
C9—C8—C7	108.7 (6)	C5—Mo—C6	98.0 (3)
C9—C8—Mo	73.4 (3)	C7—Mo—C6	35.1 (2)
C7—C8—Mo	72.1 (3)	C8—Mo—C6	57.9 (2)
C9—C8—H6	125.6	C10—Mo—C6	34.2 (3)
C7—C8—H6	125.6	C9—Mo—C6	57.7 (3)
Mo—C8—H6	120.5	C3—Mo—Pb	134.20 (17)
C10—C9—C8	107.0 (6)	C4—Mo—Pb	72.17 (16)
C10—C9—Mo	72.6 (4)	C5—Mo—Pb	71.52 (16)
C8—C9—Mo	71.8 (3)	C7—Mo—Pb	133.44 (17)
C10—C9—H7	126.5	C8—Mo—Pb	123.54 (18)
C8—C9—H7	126.5	C10—Mo—Pb	76.28 (15)
Mo—C9—H7	120.9	C9—Mo—Pb	88.82 (17)
C6—C10—C9	109.5 (6)	C6—Mo—Pb	100.61 (19)
C6—C10—Mo	73.2 (4)	O1—Pb—O2	51.85 (12)
C9—C10—Mo	72.8 (4)	O1—Pb—O2^ii^	120.53 (12)
C6—C10—H8	125.2	O2—Pb—O2^ii^	69.57 (15)
C9—C10—H8	125.2	O1—Pb—O1^i^	70.11 (13)
Mo—C10—H8	120.5	O2—Pb—O1^i^	115.60 (12)
C1—O1—Pb	94.6 (3)	O2^ii^—Pb—O1^i^	160.67 (12)
C1—O1—Pb^i^	142.5 (4)	O1—Pb—Mo	93.83 (9)
Pb—O1—Pb^i^	109.89 (13)	O2—Pb—Mo	105.64 (9)
C1—O2—Pb	93.5 (3)	O2^ii^—Pb—Mo	92.42 (9)
C1—O2—Pb^ii^	153.5 (4)	O1^i^—Pb—Mo	103.33 (8)
			
C10—C6—C7—C8	1.6 (7)	C10—C9—Mo—C7	−77.8 (4)
Mo—C6—C7—C8	65.4 (4)	C8—C9—Mo—C7	37.5 (4)
C10—C6—C7—Mo	−63.8 (5)	C10—C9—Mo—C8	−115.2 (6)
C6—C7—C8—C9	−1.3 (7)	C8—C9—Mo—C10	115.2 (6)
Mo—C7—C8—C9	64.8 (4)	C10—C9—Mo—C6	−36.2 (4)
C6—C7—C8—Mo	−66.2 (4)	C8—C9—Mo—C6	79.0 (5)
C7—C8—C9—C10	0.6 (7)	C10—C9—Mo—Pb	67.2 (4)
Mo—C8—C9—C10	64.6 (4)	C8—C9—Mo—Pb	−177.6 (4)
C7—C8—C9—Mo	−64.0 (4)	C10—C6—Mo—C3	159.8 (4)
C7—C6—C10—C9	−1.3 (7)	C7—C6—Mo—C3	43.1 (5)
Mo—C6—C10—C9	−64.2 (5)	C10—C6—Mo—C4	24.9 (8)
C7—C6—C10—Mo	62.9 (4)	C7—C6—Mo—C4	−91.8 (7)
C8—C9—C10—C6	0.4 (7)	C10—C6—Mo—C5	−117.7 (4)
Mo—C9—C10—C6	64.5 (5)	C7—C6—Mo—C5	125.6 (4)
C8—C9—C10—Mo	−64.0 (4)	C10—C6—Mo—C7	116.7 (6)
O2—C1—O1—Pb	1.3 (6)	C10—C6—Mo—C8	78.0 (4)
C2—C1—O1—Pb	179.4 (6)	C7—C6—Mo—C8	−38.7 (4)
O2—C1—O1—Pb^i^	−130.3 (5)	C7—C6—Mo—C10	−116.7 (6)
C2—C1—O1—Pb^i^	47.8 (9)	C10—C6—Mo—C9	36.6 (4)
O1—C1—O2—Pb	−1.3 (6)	C7—C6—Mo—C9	−80.1 (4)
C2—C1—O2—Pb	−179.3 (6)	C10—C6—Mo—Pb	−45.1 (4)
O1—C1—O2—Pb^ii^	−156.3 (5)	C7—C6—Mo—Pb	−161.8 (4)
C2—C1—O2—Pb^ii^	25.6 (13)	C1—O1—Pb—O2	−0.7 (3)
C6—C7—Mo—C3	−143.0 (4)	Pb^i^—O1—Pb—O2	150.3 (2)
C8—C7—Mo—C3	103.0 (4)	C1—O1—Pb—O2^ii^	11.1 (4)
C6—C7—Mo—C4	141.2 (4)	Pb^i^—O1—Pb—O2^ii^	162.11 (12)
C8—C7—Mo—C4	27.3 (6)	C1—O1—Pb—O1^i^	−151.0 (4)
C6—C7—Mo—C5	−64.1 (5)	Pb^i^—O1—Pb—O1^i^	0.0
C8—C7—Mo—C5	−178.0 (4)	C1—O1—Pb—Mo	106.2 (3)
C6—C7—Mo—C8	113.9 (6)	Pb^i^—O1—Pb—Mo	−102.80 (11)
C6—C7—Mo—C10	36.3 (4)	C1—O2—Pb—O1	0.7 (3)
C8—C7—Mo—C10	−77.6 (4)	Pb^ii^—O2—Pb—O1	169.1 (2)
C6—C7—Mo—C9	77.2 (4)	C1—O2—Pb—O2^ii^	−168.4 (4)
C8—C7—Mo—C9	−36.7 (4)	Pb^ii^—O2—Pb—O2^ii^	0.0
C8—C7—Mo—C6	−113.9 (6)	C1—O2—Pb—O1^i^	31.9 (4)
C6—C7—Mo—Pb	24.9 (5)	Pb^ii^—O2—Pb—O1^i^	−159.73 (13)
C8—C7—Mo—Pb	−89.0 (4)	C1—O2—Pb—Mo	−81.7 (3)
C9—C8—Mo—C3	163.5 (4)	Pb^ii^—O2—Pb—Mo	86.71 (13)
C7—C8—Mo—C3	−79.9 (4)	C3—Mo—Pb—O1	−4.2 (3)
C9—C8—Mo—C4	81.7 (4)	C4—Mo—Pb—O1	50.95 (18)
C7—C8—Mo—C4	−161.7 (4)	C5—Mo—Pb—O1	−58.0 (2)
C9—C8—Mo—C5	−112.8 (6)	C7—Mo—Pb—O1	−167.3 (3)
C7—C8—Mo—C5	3.7 (7)	C8—Mo—Pb—O1	148.6 (2)
C9—C8—Mo—C7	−116.6 (6)	C10—Mo—Pb—O1	−177.2 (2)
C9—C8—Mo—C10	−37.5 (4)	C9—Mo—Pb—O1	150.2 (2)
C7—C8—Mo—C10	79.0 (4)	C6—Mo—Pb—O1	−153.0 (2)
C7—C8—Mo—C9	116.6 (6)	C3—Mo—Pb—O2	47.2 (3)
C9—C8—Mo—C6	−78.2 (5)	C4—Mo—Pb—O2	102.33 (19)
C7—C8—Mo—C6	38.3 (4)	C5—Mo—Pb—O2	−6.7 (2)
C9—C8—Mo—Pb	2.9 (5)	C7—Mo—Pb—O2	−115.9 (3)
C7—C8—Mo—Pb	119.4 (4)	C8—Mo—Pb—O2	−160.1 (2)
C6—C10—Mo—C3	−36.1 (7)	C10—Mo—Pb—O2	−125.9 (2)
C9—C10—Mo—C3	81.2 (7)	C9—Mo—Pb—O2	−158.4 (2)
C6—C10—Mo—C4	−168.1 (4)	C6—Mo—Pb—O2	−101.7 (2)
C9—C10—Mo—C4	−50.7 (5)	C3—Mo—Pb—O2^ii^	116.6 (3)
C6—C10—Mo—C5	71.0 (4)	C4—Mo—Pb—O2^ii^	171.78 (18)
C9—C10—Mo—C5	−171.7 (4)	C5—Mo—Pb—O2^ii^	62.8 (2)
C6—C10—Mo—C7	−37.3 (4)	C7—Mo—Pb—O2^ii^	−46.5 (3)
C9—C10—Mo—C7	80.0 (4)	C8—Mo—Pb—O2^ii^	−90.6 (2)
C6—C10—Mo—C8	−79.6 (4)	C10—Mo—Pb—O2^ii^	−56.4 (2)
C9—C10—Mo—C8	37.7 (4)	C9—Mo—Pb—O2^ii^	−89.0 (2)
C6—C10—Mo—C9	−117.3 (6)	C6—Mo—Pb—O2^ii^	−32.22 (19)
C9—C10—Mo—C6	117.3 (6)	C3—Mo—Pb—O1^i^	−74.7 (3)
C6—C10—Mo—Pb	134.2 (4)	C4—Mo—Pb—O1^i^	−19.50 (18)
C9—C10—Mo—Pb	−108.5 (4)	C5—Mo—Pb—O1^i^	−128.5 (2)
C10—C9—Mo—C3	−137.2 (4)	C7—Mo—Pb—O1^i^	122.2 (3)
C8—C9—Mo—C3	−22.0 (6)	C8—Mo—Pb—O1^i^	78.1 (2)
C10—C9—Mo—C4	138.9 (4)	C10—Mo—Pb—O1^i^	112.3 (2)
C8—C9—Mo—C4	−105.8 (4)	C9—Mo—Pb—O1^i^	79.7 (2)
C10—C9—Mo—C5	13.8 (7)	C6—Mo—Pb—O1^i^	136.50 (19)
C8—C9—Mo—C5	129.1 (5)		

Symmetry codes: (i) −*x*+1, −*y*+1, −*z*+1; (ii) −*x*, −*y*+1, −*z*+1.
